# Understanding the Adhesion Mechanism of Hydroxyapatite-Binding Peptide

**DOI:** 10.1021/acs.langmuir.1c02293

**Published:** 2022-01-07

**Authors:** Tal Duanis-Assaf, Tan Hu, Maayan Lavie, Zhuo Zhang, Meital Reches

**Affiliations:** † Institute of Chemistry and The Center for Nanoscience and Nanotechnology, The Hebrew University of Jerusalem, Jerusalem 91904, Israel; ‡ College of Food Science and Technology, Huazhong Agricultural University, Wuhan, Hubei 430070, People’s Republic of China; § Key Laboratory of Environment Correlative Dietology, Huazhong Agricultural University, Ministry of Education, Wuhan, Hubei 430070, People’s Republic of China

## Abstract

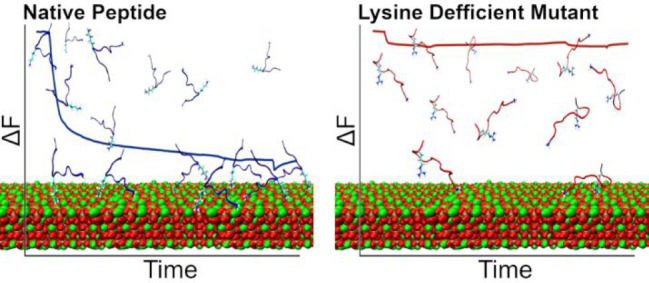

Understanding the interactions between the protein collagen and hydroxyapatite is of high importance for understanding biomineralization and bone formation. Here, we undertook a reductionist approach and studied the interactions between a short peptide and hydroxyapatite. The peptide was selected from a phage-display library for its high affinity to hydroxyapatite. To study its interactions with hydroxyapatite, we performed an alanine scan to determine the contribution of each residue. The interactions of the different peptide derivatives were studied using a quartz crystal microbalance with dissipation monitoring and with single-molecule force spectroscopy by atomic force microscopy. Our results suggest that the peptide binds via electrostatic interactions between cationic moieties of the peptide and the negatively charged groups on the crystal surface. Furthermore, our findings show that cationic residues have a crucial role in binding. Using molecular dynamics simulations, we show that the peptide structure is a contributing factor to the adhesion mechanism. These results suggest that even small conformational changes can have a significant effect on peptide adhesion. We suggest that a bent structure of the peptide allows it to strongly bind hydroxyapatite. The results presented in this study improve our understanding of peptide adhesion to hydroxyapatite. On top of physical interactions between the peptide and the surface, peptide structure contributes to adhesion. Unveiling these processes contributes to our understanding of more complex biological systems. Furthermore, it may help in the design of de novo peptides to be used as functional groups for modifying the surface of hydroxyapatite.

## Introduction

Bones and dental tissues are composite materials that comprise an organic phase of mainly collagen type I fibrils and an inorganic phase of hydroxyapatite (HAp) crystals.^1−3^ Like many other biocrystals, the remarkable mechanical properties of bones are achieved by their complex and hierarchical structure.^4−6^ The process of biomineralization is predominantly thought to be a nonclassical crystallization pathway. This process consists of multiple stages during which several intermediate calcium phosphate species are formed, leading to the formation of crystalline HAp.^7−9^ This process is kinetically favorable to classical crystal nucleation,^7^ and its precursors are thermodynamically stable.^10^ However, the process is not fully understood.^11^

Studies in recent years have shown that both collagen^12,13^ and noncollagenous proteins^14^ contribute to the biomineralization of HAp. It was previously established that acidic residues (i.e., glutamic acid and aspartic acid) promote HAp mineralization by binding calcium ions.^14,15^ It was also shown that such residues can stabilize other biominerals and their precursors such as amorphous calcium carbonate.^16^ Recently, Wang et al.^17^ were able to induce HAp mineralization on the surface of a gold substrate coated with self-assembled monolayers of alkanethiols featuring acidic oligopeptides.

Short peptides are frequently used as model systems to study this process. For this purpose, phage display is commonly used to identify HAp-binding peptide sequences.^18−20^ Chung et al.^20^ investigated HAp-binding peptides derived from phage display and found that species with high affinity contain numerous hydroxylated residues. They revealed that collagen and other bone-related proteins are rich in hydroxylated residues domains. Using molecular dynamics (MD), they showed that the peptide sequences adhere via hydrogen bonds between hydroxyl side groups and phosphate moieties in the substrate. Furthermore, they determined that the distances between adjacent hydroxyl groups were similar to those in collagen domains. These distances were correlated to the distance between phosphate groups in the (100) crystal face of HAp. A recent MD study supports these claims by showing the interactions of several amino acid side chains with the surfaces of HAp (100) and (001).^21^

Sahai and her collaborators^11,22^ studied the interactions between a cationic peptide identified using a phage-display library and three derivative sequences featuring different net charges. They used MD simulations, mass depletion, and circular dichroism (CD) and showed that the peptide net charge has the highest contribution to the interaction with HAp. In contrast, Gungormus et al.^23^ showed that adjacent oppositely charged residues improve the mineralization rate. Their results indicate that the net charge of the peptide was in fact less important than the number of repeating oppositely charged residue pairs. These seemingly contradicting results emphasize the complexity of these systems. Moreover, it was previously shown that conformation has a role in the interaction with solid surfaces.^23−25^

Roy et al.^26^ used phage display and quartz crystal microbalance with dissipation monitoring (QCM-D) to find HAp-binding peptide sequences and showed that a 12-mer peptide with the sequence SVSVGMKPSPRP adheres to HAp with high affinity. By comparing to protein databases, they showed that this peptide comprises two sections, which are related to bacterial phosphate-binding enzymes.^26^ Weiger et al.^27^ used surface plasmon resonance (SPR) analysis and showed that the SVSV moiety binds HAp while the remaining sequence adheres less potently. They suggested that the SVSV subset of the peptide was the binding site and that the remainder of the sequence has a structural role that stabilizes the interaction. While it is known that this peptide has a strong affinity to HAp, the adhesion mechanism remains unclear. Moreover, this sequence contains several hydroxylated residues (serine) and a relatively high net charge of +2, making it an ideal model HAp binding peptide.

In this work, we used QCM-D and single-molecule force spectroscopy (SMFS) using an atomic force microscope (AFM) to measure the adhesive interactions between the SVSVGMKPSPRP peptide and HAp surface. By combining these quantitative experimental methods with an alanine scan, we were able to show that lysine in the seventh position has a role in stabilizing the interaction with HAp. Moreover, using Fourier-transform infrared spectroscopy (FT-IR) along with MD simulations, we were able to deduce how the peptide secondary structure takes part in the adhesion mechanism.

## Results and Discussion

The adhesion of the native peptide, SVSVGMKPSPRP (Table 1), to HAp was measured using QCM-D. The peptide solution was circulated in a flow cell over a commercially available QCM-D sensor coated with HAp nanoparticles. The process was monitored for 18 h in a buffered solution (pH 7.2, and physiological ionic strength of 154 mM) at room temperature. As HAp comprises calcium phosphate, all measurements were performed in TRIS buffer rather than phosphate-buffered saline (PBS) to avoid possible interactions between the peptide and phosphate ions in the solution.

**Table 1:**
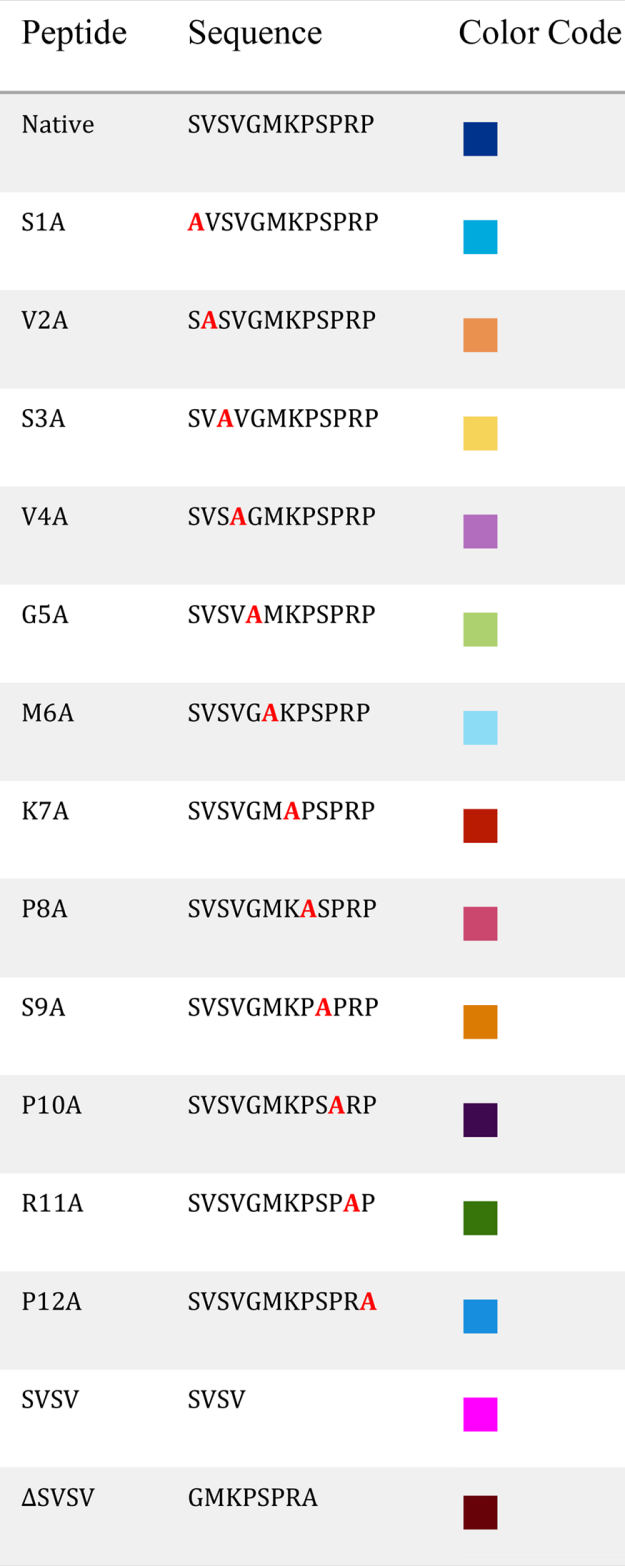
Studied Peptide Sequences^a^

a The native peptide sequence and its derivatives including two subdomains (SVSV and ΔSVSV) and 12 alanine scan derivatives are sequentially denoted XNA, where N and X are the positions and one-letter abbreviation of the amino acid replaced by alanine, respectively. The color code is used to distinguish between the different peptides in all of the figures.

**Figure 1:**
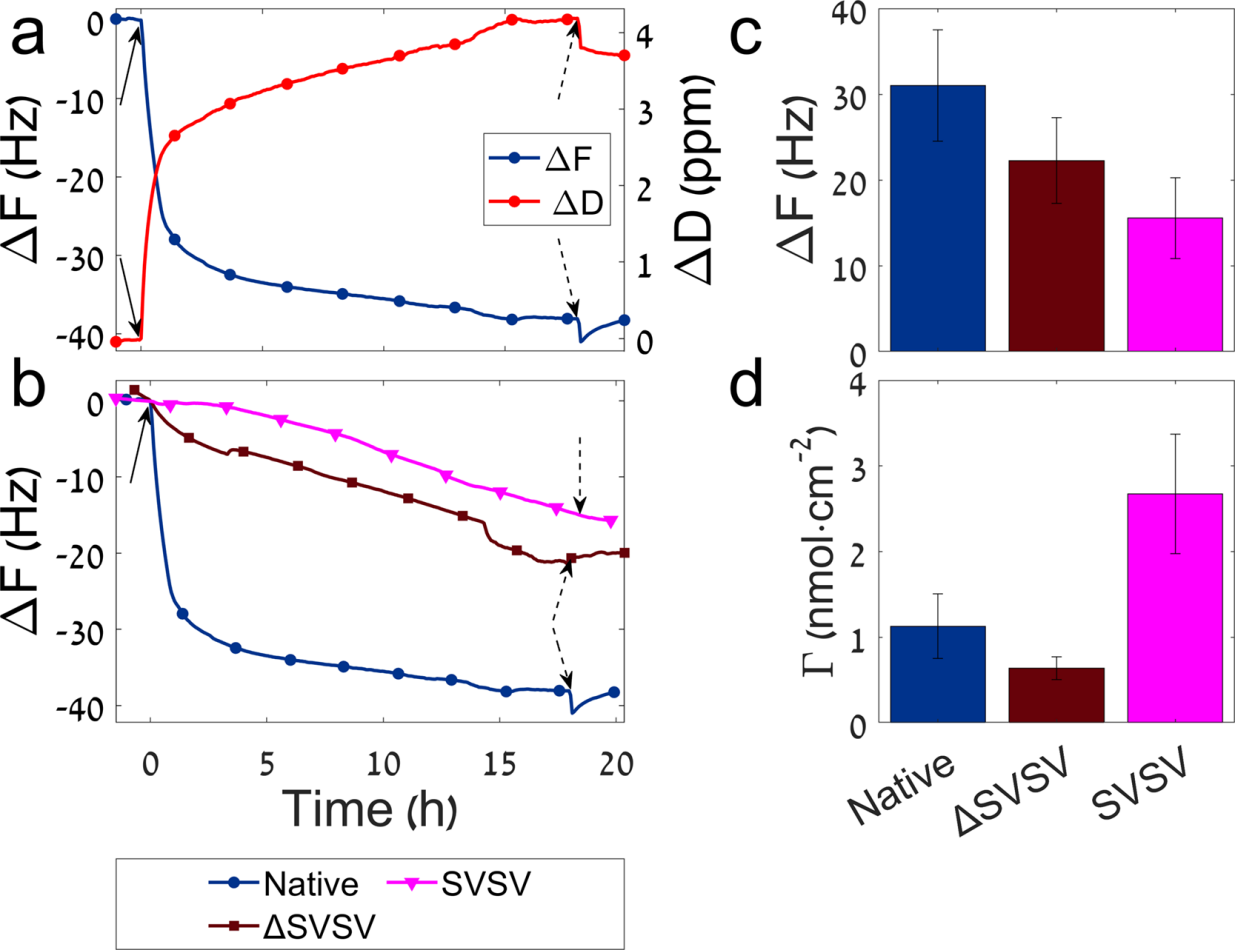
Comparison between the adhesion of the native peptide and SVSV derivatives to HAp using QCM-D analysis. (a) A typical adhesion curve of the native peptide. The blue line represents the change in measured frequency, and the red line represents the change in dissipation signal. (b) Typical adhesion curves of the native peptide, SVSV, and Δ*S*VSV sequences. (a and b) The arrows illustrate the point of injection (continuous line) and washing with buffer (dashed line). (c) The change in the measured frequency (between the point of injection and end of washing) between the native peptide and SVSV derivatives. (d) The peptide surface concentration, after the washing period, was calculated according to the Voigt mass. The error bars represent the standard error of the mean based on 2−3 repeats. Frequency data are taken from the ninth overtone.

Figure 1a shows a typical adhesion profile of the native peptide over 18 h. As the peptide adheres to the surface, the frequency decreases due to the increasing mass of the adsorbed layer, while the dissipation increases due to the formation of a viscoelastic layer.^28^ Most of the adhesion occurs during the first 2 h, as can be seen from the slope of the frequency, which gradually decreases. The process finally reaches a steady state after 15–18 h. The HAp substrate was finally rinsed with buffer after 18 h to wash components of the layer that were not well adhered to the surface. A sharp response in frequency and dissipation was commonly observed when changing between the peptide and buffer solutions. This abrupt change may be attributed to changes in the mechanical properties of the solution.^29^

To assess the activity of the two regions of the peptide, we conducted adhesion assays for two derivatives of the native peptide, SVSV and an SVSV-deficient sequence, GMKPSPRP (ΔSVSV). Figure 1b shows an overlay of the typical adhesion curves for the native peptide and both SVSV derivatives. From the adhesion curves, it seems that both derivatives had a lower frequency change when compared to the native peptide. The native peptide showed an average reduction of 31 ± 6 Hz, whereas SVSV and ΔSVSV showed a reduction of 16 ± 5 and 22 ± 5 Hz, respectively (Figure 1c). However, the change in frequency correlates with the added mass.

To compare the added mass, the curves were fit with the Voigt viscoelastic model.^30,31^ Similar to the frequency and dissipation, the area density obtained from the Voigt fitting also reflected the abrupt changes observed when changing the media between baseline, peptide, and washing solutions. The change in the area density was therefore calculated separately for every stage (i.e., layer buildup and washing), and the overall area density for each experiment was evaluated by summing the value for both stages (Figure S1). Finally, to account for the different molecular weights of the adhered peptides, a molar surface concentration was obtained by dividing the area density by the molecular weight of the corresponding peptide. It is worth mentioning that, due to film hydration, a considerable portion of the mass could probably be attributed to added water. However, due to the lack of an accurate estimate of water uptake, and under the assumption that the water/peptide ratio is similar in all measurements, the water content was not accounted for when calculating the surface concentration. Moreover, when calculating the surface concentration of the peptide, the surface roughness and morphology are a concern.^32,33^ The surface morphology of the QCM-D sensor was analyzed using AFM before and after adhesion measurement of the native peptide (Figure S2). The morphology of the peptide layer seems to build up on existing morphological features, and an increase of roughly 10% in the roughness of the sensor was observed (Table S1). This increased roughness could lead to trapped water^32^ and increased surface area, both of which may cause an overestimation of the peptide surface concentration. Nevertheless, the surface concentration should still act as a reliable measure of the amount of adhered peptide. Figure 1d shows the summary of these measurements of surface concentration. Remarkably, SVSV showed notably higher adhesion than the native peptide in terms of surface concentration, with 2.7 ± 0.7 nmol cm^–2^ for SVSV and 1.1 ± 0.4 nmol cm^–2^ for the native peptide. This may support the thesis by Becker and collaborators^27^ who suggested that this short peptide is the active site of the native peptide. Even so, neither of these peptides’ overall surface concentration was significantly different from that of the native peptide as determined by one-way ANOVA with a *p*-value of 0.05. Moreover, the adhesion of SVSV was markedly slower and did not seem to reach a steady-state over the entire 18 h measurement. This slower adsorption rate may suggest a different adhesion mechanism.^34^ Moreover, the higher surface concentration could be attributed to the smaller size of the molecule.

**Figure 2:**
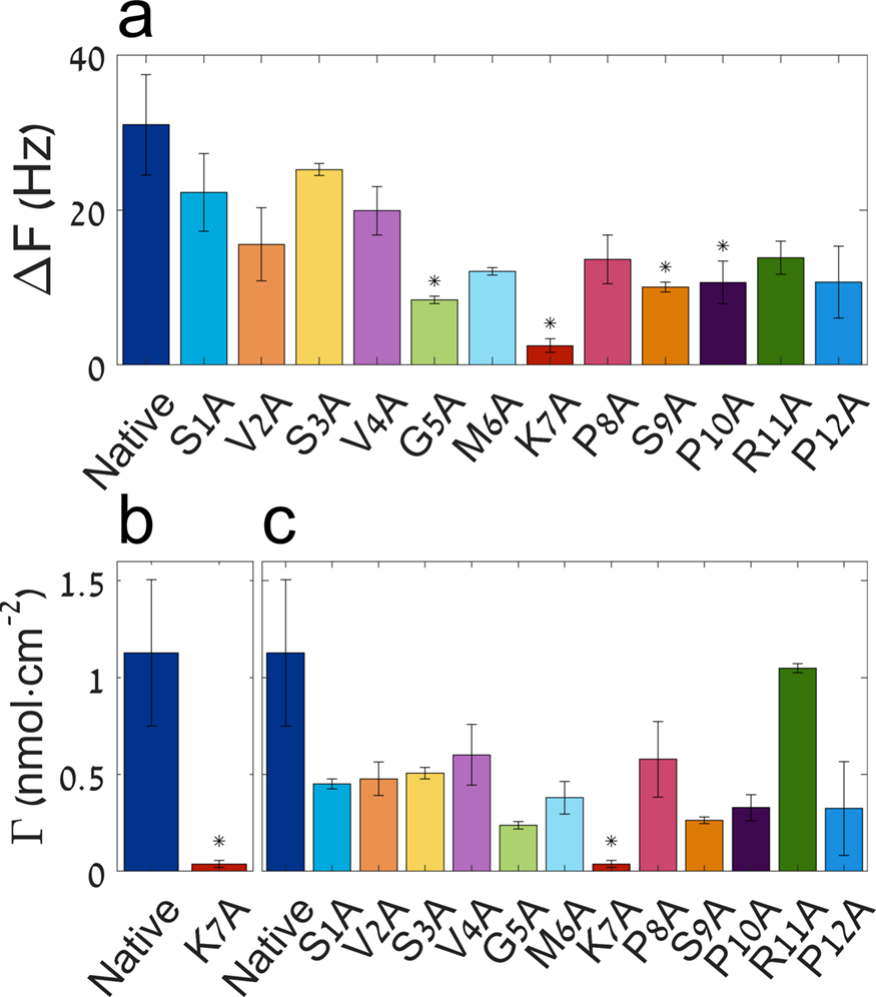
A comparison between the adhesion of the different peptides to HAp monitored by QCM-D. (a) A comparison of the measured frequency change between the native peptide and all alanine scan derivatives. (b and c) Peptide surface concentration after the washing period, calculated according to Voigt mass. (b) A comparison between the native peptide and K7A. (c) A comparison between the native peptide and all alanine scan derivatives. The error bars represent the standard error of the mean based on 2−3 measurements. The asterisks represent significantly different mean values in comparison to the native peptide as determined by one-way ANOVA followed by the post hoc Tukey test.

To determine which residue is crucial for the adhesion, we conducted a full alanine scan. We used a library of peptide derivatives as described in Table 1. Typical QCM-D unsmoothed adhesion curves of all derivatives are shown in Figure S3. Figure 2 summarizes the frequency difference measured for each peptide and the surface concentration, averaged over 2−3 repeats. The surface concentration was calculated according to the Voigt mass and molecular weight. Interestingly, when substituting lysine at position 7 with alanine (K7A), the overall frequency difference was reduced by an order of magnitude (Figure 2a), from 31 ± 6 Hz for the native peptide to 2.5 ± 0.9 Hz for K7A. The surface concentration was further reduced by 2 orders of magnitude (Figure 2b), from 1.1 ± 0.4 nmol cm^–2^ in the native peptide to 0.04 ± 0.02 nmol cm^–2^ in K7A. This significant reduction in adhesion suggests that this residue has an important role in the interaction with the HAp surface.

**Figure 3:**
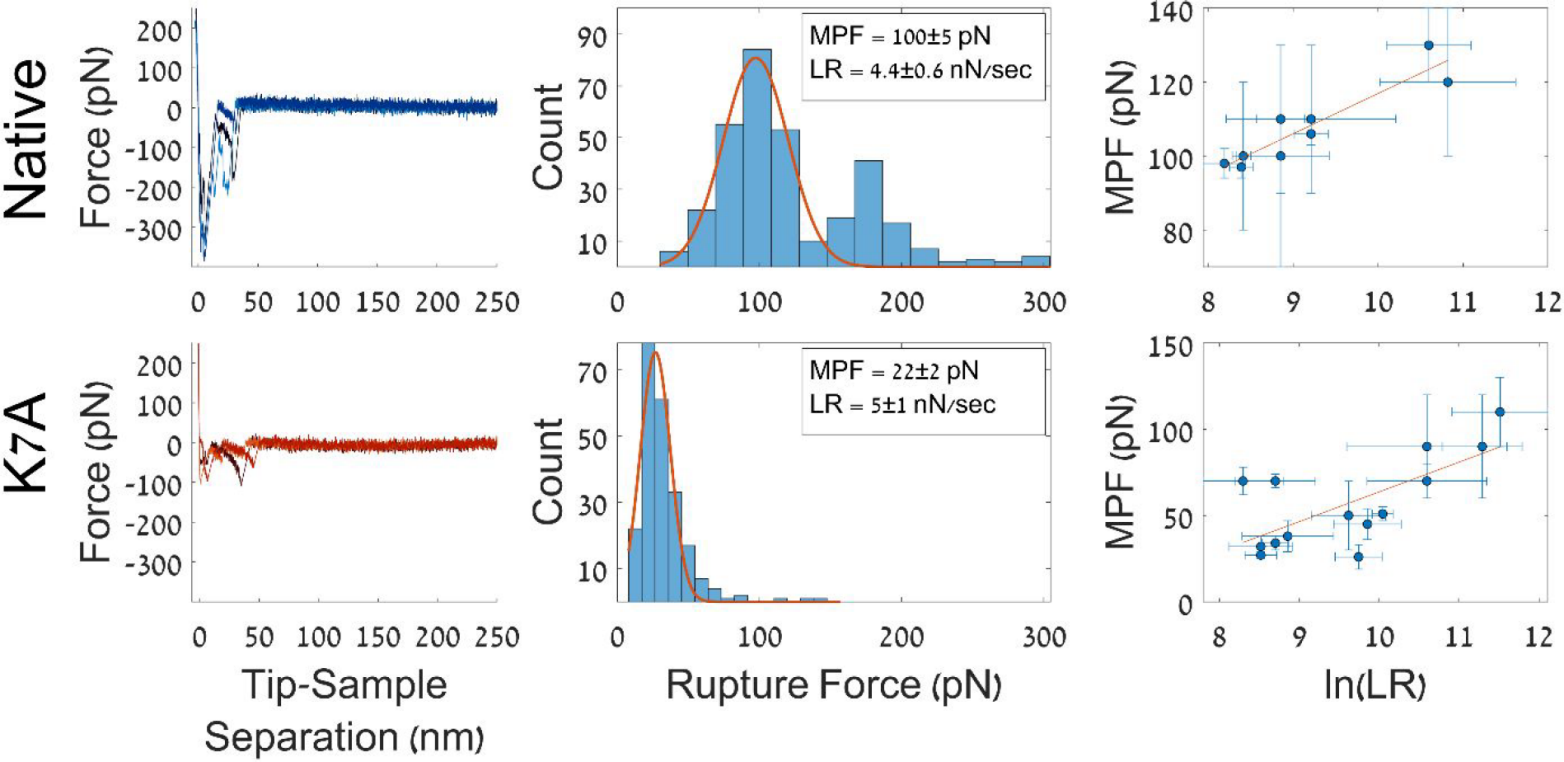
Adhesion force measurements using SMFS. The top panel shows details about the native peptide, and the bottom panel shows details about the K7A derivative. (left) Typical force profiles of the adhesion interaction. (middle) Unbinding force histograms with the calculated most probable force (MPF) and average loading rate (LR). The errors are confidence intervals calculated for α = 0.05. (right) Bell–Evans plots.

To further investigate the contribution of lysine to the interaction with HAp, we performed SMFS analysis using an AFM. Recently, force spectroscopy was used to study how peptides, proteins, and even single amino acids interact in different systems, including ligand–receptor interactions,^35−37^ chiral induced spin exchange,^38,39^ and adhesion to solid surfaces.^24,40−43^ We coupled peptide molecules to an AFM tip and performed an adhesion assay against a polycrystalline HAp surface. In this experiment, the probe was approached to the substrate to allow the peptide to adhere, and the tip was then retracted until full separation was obtained. Typical force profiles of both the native peptide and K7A are shown in Figure 3.

SMFS experiments were performed at different scanner speeds, and each measurement was repeated at least 4000 times. The data were analyzed using ForSDAT^44^ (see Supporting Information ForSDAT configuration files). Briefly, the unbinding force was calculated using the worm-like chain (WLC) model, and specific interactions were determined using the smoothing peak correlation method.^44^ As an additional precaution, experiments featuring an unusually low number of specific interactions were excluded from further analysis. The cutoff values used for the native and K7A peptides were 4% and 2.5%, respectively. The overall frequency of specific interactions observed for K7A was 3.6% ± 0.6%; this value was substantially lower than that of the native peptide, 8% ± 1%, possibly due to the lower bond stability. The lower cutoff frequency used for K7A was meant to account for this overall lower frequency of specific interaction occurrence.

The most probable force (MPF) for each experiment was extracted by fitting the histogram with a Gaussian. In some experiments, the unbinding forces feature a bimodal distribution, such as in the case of the native peptide histogram in Figure 3 (top middle pane). This may be due to coexisting bonds breaking apart nearly simultaneously, resulting in unresolved force peaks.^45^ To overcome this, for these data, the MPF was evaluated by fitting a bimodal Gaussian, and the peak with the lower force was considered the MPF.

For the pair of histograms shown in Figure 3, the MPF of the native peptide was roughly 5 times higher than that of K7A. However, the unbinding force deduced from SMFS measurements depends on the loading rate.^46,47^ Therefore, to compare the adhesive interactions of the two peptides, the kinetic parameters of the interactions between the bound peptides and HAp surface were determined using the Bell, Evans, and Ritchie model.^46,47^ This commonly used model depicts a logarithmic relation between the loading rate (*r*) and interaction force (*F*_r_) as follows:
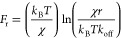
1where *k*_B_ is the Boltzmann constant, *K*_off_ is the dissociation rate, χ is the distance of the transition state along the axis of applied force, and the temperature *T* was assumed to be 298 K. The Bell, Evans, and Ritchie plots for both peptides are shown in Figure 3.

**Figure 4:**
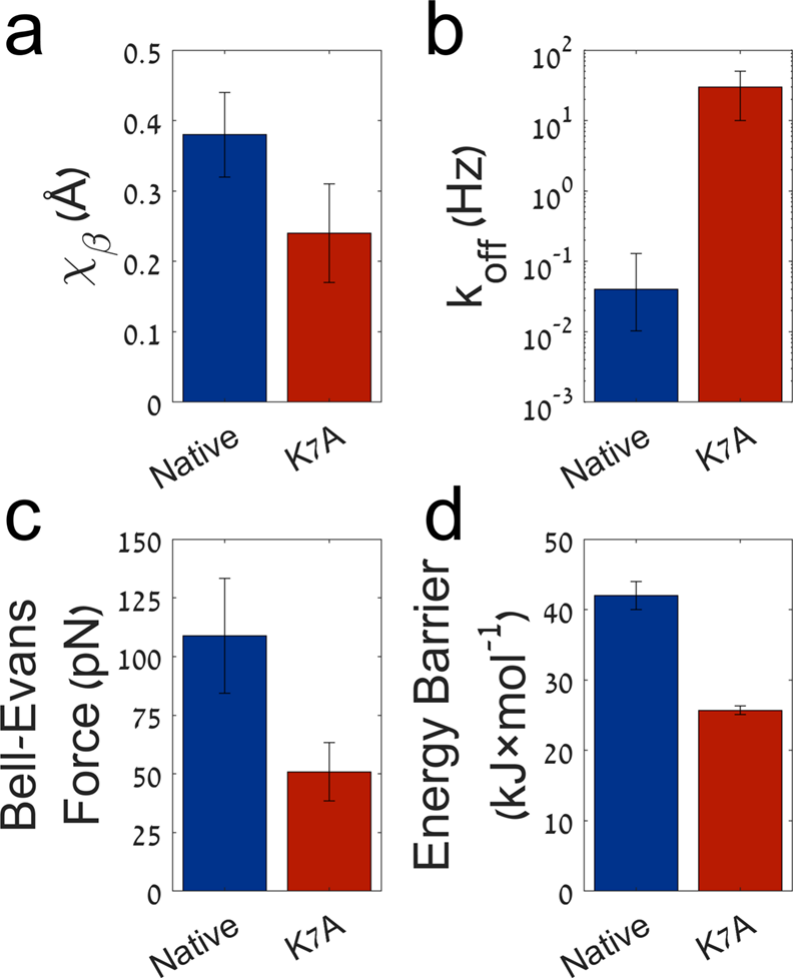
Kinetic parameters of the interaction between HAp and the native peptide and K7A derivative were calculated using the Bell–Evans model. (a) The transition state distance (χ_β_). (b) The bond dissociation rate (*k*_off_). (c) The rupture force was calculated according to the Bell–Evans model using the kinetic parameters (χ_β_, *k*_off_) at a loading rate of 10 nN s^–1^. (d) The dissociation energy barrier. The error bars represent the standard error of the mean.

A summary of the kinetic parameters is shown in Figure 4. χ was slightly higher for the native peptide as compared to K7A, 0.38 ± 0.06 and 0.24 ± 0.07 Å, respectively, suggesting the unbinding of K7A occurs at a shorter distance. However, it seems to be within the range of the standard error. The dissociation rate of K7A was 3 orders of magnitude greater than that of the native peptide, meaning the lifetime of the interaction is markedly shorter. To compare the interaction force, a theoretical value was calculated using eq 1 for a loading rate of 10 nN s^–1^. The theoretical interaction force of the native peptide, 110 ± 20 pN, was more than 2-fold higher than that of K7A, 50 ± 10 pN. Eventually, the dissociation barrier energy was calculated using the relation:
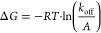
2where Δ*G* is the energy barrier, *R* is the gas constant, *T* is the temperature, *k*_off_ is the dissociation rate, and *A* is the Arrhenius prefactor of frequency. We used an *A* value of 10^6^ Hz^41,48^ and a temperature of 298 K. The energy barrier calculated for the native peptide, 42 ± 2 kJ mol^–1^, was notably higher than that of K7A, 25.7 ± 0.6 kJ mol^–1^. Taken together, these results support our QCM-D measurements, suggesting that lysine is indeed crucial for the interaction to take place.

**Figure 5:**
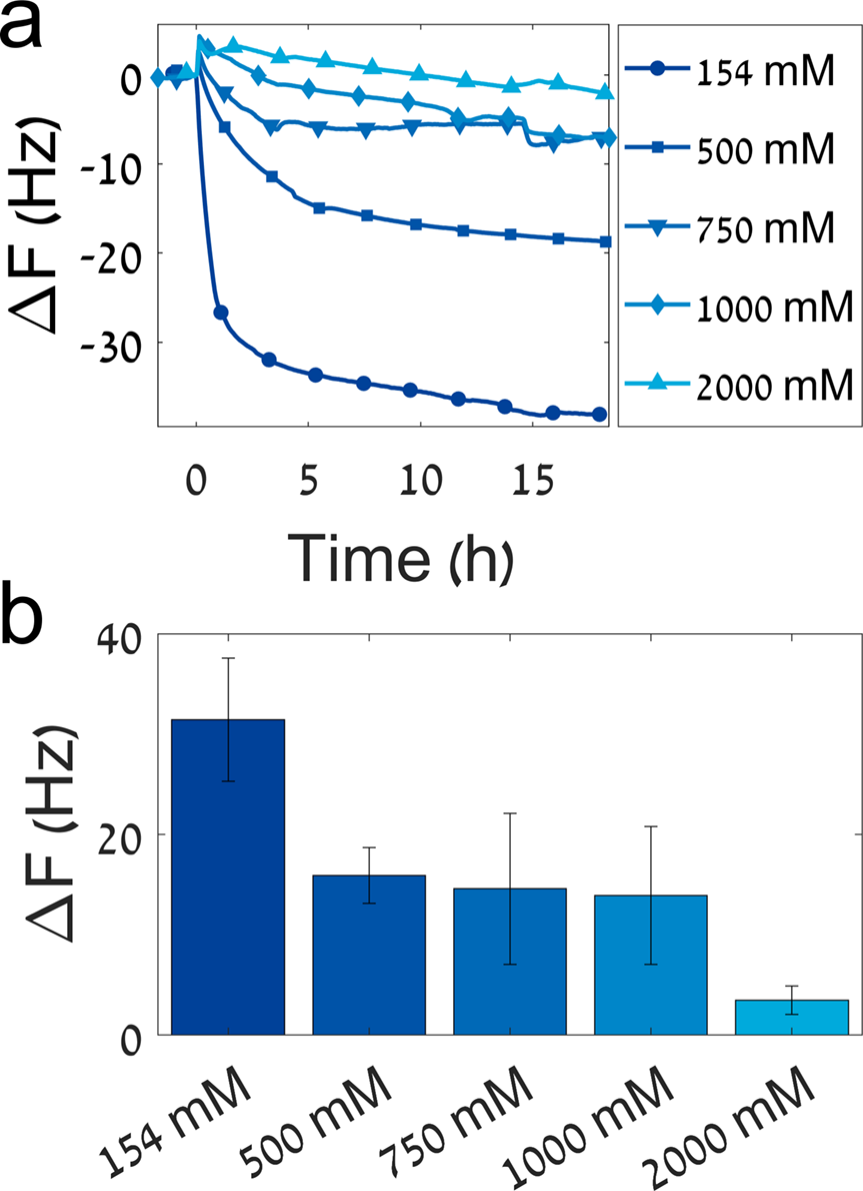
Peptide adhesion under different ionic strengths monitored by QCM-D. (a) The adhesion curves of the native peptide in increasing buffer ionic strength. (b) The change in frequency over the course of 18 h starting from the point of injection. The adhesion decreases with the increase in ionic strength. The error bars represent the standard error of the mean of 2−3 measurements.

Because lysine is charged under physiological conditions, we set out to determine whether the nature of the peptide–surface interaction is electrostatic. To assess the contribution of ionic interactions, the adhesion assay was repeated with the native peptide, under different buffer ionic strengths (Figure 5). Figure 5b shows the summary of the results, with a decrease in overall adhesion as the ionic strength increases. These results suggest that ionic interactions have an important contribution to the binding of the peptide to HAp. Nevertheless, the peptide R11A, where arginine at position 11 is replaced with alanine, did not show a significant difference from the native peptide (Figure 2c). K7A and R11A have the same net charge. Moreover, they represent the only two charged residues in the sequence. Furthermore, it was recently shown that arginine can promote the adsorption of fibronectin to HAp.^49^ Therefore, ionic interactions cannot be the only factor contributing to the interaction. The peptide structure likely has a significant part in the adhesion mechanism.

To investigate the contribution of the peptide conformation, the secondary structures of the native peptide, K7A, and R11A were analyzed using FT-IR spectroscopy. FT-IR results are presented in Figure S4, and the analysis is further described in the Supporting Information results section. These results suggest that all three peptides adopted a turn-like structure in solution. Next, attenuated total reflection (ATR) FT-IR spectroscopy was used to determine whether any structural changes occur during the adsorption to the HAp surface. For this purpose, the HAp-coated QCM-D sensors were used after the QCM-D adhesion assay with the native peptide. The results are presented in Figure S5 and further described in the Supporting Information results section. Interestingly, a possible increase in intermolecular β-sheet formation was observed. This suggests that the bound peptide molecules can interact with each other. When the measurements were performed with K7A, no clear signal was observed at the amide-I band. This is not surprising, given the poor adhesive capabilities of this peptide. Therefore, a different approach was necessary to investigate the peptide structure.

**Figure 6:**
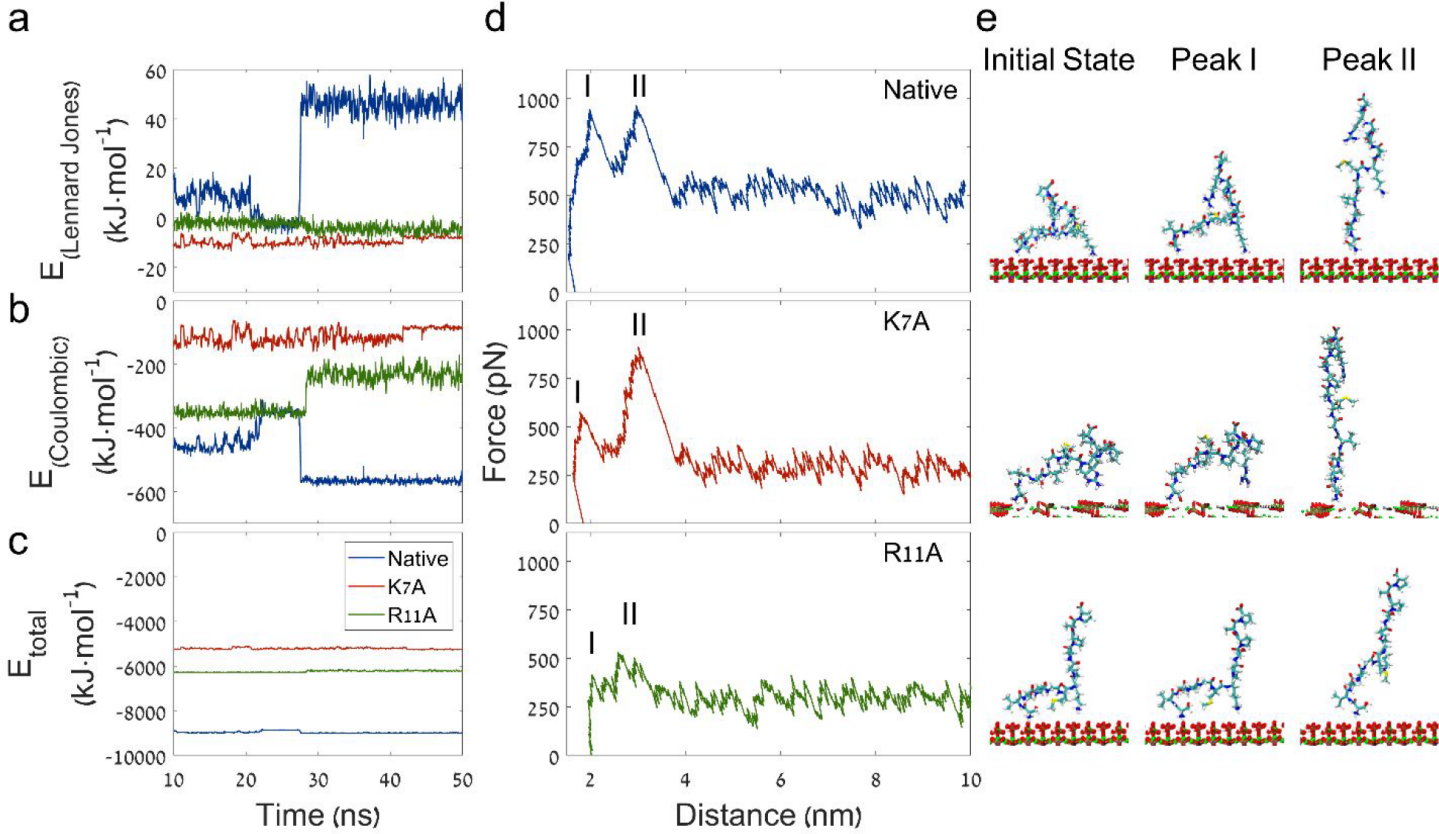
Energy, force of interaction, and peptide conformation between the native, K7A and R11A peptides and HAp (100) surface, predicted by molecular dynamics simulations. The left panel shows the magnitude of the different potentials between the peptide and the HAp surface during the first 50 ns of energy simulation. Hydrophobic interactions were calculated using the Lennard-Jones potential (a), Coulombic potential (b), and the total binding energy (c). The middle panel shows the change in force versus pull-off distance calculated using SMD simulations (d). The right panel shows snapshots of peptide conformations taken at the beginning of the SMD simulation (initial state) and two force peaks marked I and II on the corresponding SMD force versus distance curve.

To investigate the conformation adopted by the different peptides, while binding the HAp surface, MD simulations were performed. First, the binding energies of the native, K7A, and R11A peptides were calculated and monitored over a period of 50 ns. Figure 6 shows the binding energies based on the Lennard-Jones potential, that is, the short-ranged van der Waals interactions (a), the short-ranged Coulombic interactions (b), and the total binding energy (c) for these three peptides over the last 40 ns of simulation. The energies of the systems for the first 10 ns of simulations were omitted due to equilibrium. It was clear that all three peptides reached stable states on the HAp surface during the simulations as indicated by the plateau profiles of the total binding energies. The Coulombic potential energy had approximately 10-fold greater magnitude than the Lennard-Jones potential energy, supporting our finding that the interaction is electrostatic in nature. The electrostatic interactions between the native peptide and the HAp surface markedly increased within the first 30 ns, and the repulsions from the van der Waals forces were also increased. It was interesting to find that R11A interacted with HAp through electrostatic attractions that were somehow disturbed as the peptide bound to the surface. However, as K7A approached the surface, it was found that the binding was favored by both van der Waals and electrostatic interactions, as both of the potentials had negative values.

All three peptides exhibited a negative total binding energy with the HAp surface. This indicates the bound state was energetically preferable. However, the native peptide’s total binding energy had the highest magnitude, suggesting it has the highest affinity. R11A interacted to a lesser extent with HAp as compared to the native peptide but had stronger Coulombic interactions and a higher magnitude of the total binding energy as compared to K7A. These results reside well with our experimental results, which showed a similar trend, with the native peptide adhering the most and K7A the least.

At the end of the MD simulation, all three peptide derivatives adopted a turn-like structure that exposed two cationic moieties to the substrate, as shown in Figure 6e (initial state) and Figures S6–S8. The native and R11A peptides adhered to the surface via the N-terminus as well as the side chain of lysine. Lacking lysine, K7A interacted via the side chain of arginine instead, while adopting a different structure to accommodate the second binding site.

Next, steered molecular dynamics simulations (SMD) were performed to monitor the interaction of individual residues with the surface. In this simulation, the molecule is pulled off of the surface by applying a constant pulling force on the molecule’s center of mass (COM) in resemblance to the SMFS experiment.^22^ It is worth mentioning that this simulation does not match the exact experimental conditions, where the pulling force is applied on the C-terminus, and the molecule is chemically bound to a linker. Nonetheless, we believe the simulation shares the essence of the SMFS experiment. Thus, the improved resolution provided by the simulation allows resolving which of the residues interacts with the surface. Figure 6d shows the simulated SMD force versus the distance pull-off curves for the native peptide, K7A, and R11A. Figures 6e and S6–S8 show snapshots of the peptide conformation at the initial state, as were fed into the SMD simulation (the conformation obtained at the end of the MD simulation), and at the time corresponding to the first and second force peaks marked on the SMD curves as I and II, respectively. Each force peak corresponds to a rupture event, followed by considerable movement of the COM along the direction of pulling force. The first rupture event corresponds to the detachment of either lysine (native and R11A) or arginine (K7A) side chains. After the first rupture event, all peptides underwent a considerable conformational change due to the applied external force, eventually losing their turn-like structure, and adopting an almost completely stretched backbone. After backbone stretching, the N-terminus was detached during the second rupture event. Following the second rupture event, the molecule was freely pulled away from the surface (see Supporting Information videos S1–S3). Interestingly, during the entire course of the SMD simulation, the force between the peptide and HAp surface did not reach baseline values, indicating some long-range electrostatic interactions were present in the simulation. These results can also explain the adhesiveness of the SVSV derivative, which can also interact via the N-terminus.

To more accurately evaluate the secondary structure that each peptide adopted, DSSP^2^ analysis was employed. The number of residues contributing to each secondary structure element throughout the MD simulations was calculated (Figure S9). All three peptides featured mostly unstructured domains and some turn-like structures. The native peptide seemed slightly more structured, featuring more turn-like structures than the other derivatives. A similar tendency to form turns was observed using FT-IR as well. Similarly, even though studying a different system, Mirau et al.^25^ showed that during adhesion their peptide adopted the secondary structure of a turn. Interestingly, both K7A and R11A had roughly 25% turn-like structures. However, when examining the locations of these features (Table S3), we determined that the turn-like structure in K7A was slightly shifted toward the C-terminus. This could be due to the peptide binding to the surface via the arginine next to the C-terminus.

The turn-like structure divides the sequence into two arms. In the native and R11A peptides, one arm is strongly bound to the HAp surface via the Lys7 side chain and the N-terminus. The second arm, Ser9 through Pro12, is more free to twist and bend. In K7A, the shifted position of the turn-like structure also led to a shorter unstructured domain, forming the free arm of the peptide. In this derivative, the free arm was only 3 amino acids long. Moreover, one of these residues, arginine, was bound to the surface. The free arm of the peptide may interact with the solvent or with other peptide molecules adhered to the crystal surface. Such interactions may contribute to the stability of the bound molecule, thus improving the adhesive nature of the peptide. This can also explain the increased peak at 1625 observed for the native peptide using ATR FT-IR, while adsorbed to the surface. This peak was previously correlated to the formation of intermolecular β-sheets.^50^

By and large, our results support previous works that showed that Coulombic interactions dominate the adhesion of HAp binding peptides.^11,22,23^ However, we show here that even small structural changes may have a striking impact on adhesion.

Taken together, these results shed some light on the nature of the adhesion process. We suggest a simple secondary structure-driven mechanism, which maximizes the stability of peptide binding via electrostatic interactions. The peptide adopts a curved structure in solution. This structure exposes two cationic groups, the N-terminus and Lys7 side group, to adhere to the negatively charged (100) surface. Once adhered, the peptide has a free arm that may interact with the solvent or with other molecules adsorbed on the surface. These interactions may have a stabilizing effect, thus strengthening the interaction and improving the overall adhesion.

## Conclusion

We found using QCM-D and SMFS combined with alanine scan that the interaction between SVSVGMKPSPRP and HAp crystals is predominantly electrostatic. Moreover, the presence of a cationic residue at position 7 has a crucial role in mediating the adhesion. By combining this with MD and SMD simulations, we were able to infer how the secondary structure of the peptide could take part in the adhesion process. Our results suggest that even small changes to the peptide sequence, leading to small shifts in the secondary structure, could lead to a significant reduction in adhesion.

An alanine scan provided valuable information for identifying the binding sites. The important role of a cationic residue in the seventh position was observed. Nevertheless, in the context of this work, this method also raises the question whether other charged residues, both positive and negative, could serve a role similar to that of lysine. Such screening methodologies could improve our understanding of the nature of peptide and protein adhesion processes. Finally, the findings presented in this work may help to design new peptides that can be used as functional groups on top of HAp.

## Experimental Section

### Materials

AFM probes with silicone tips (MSNL10) were purchased from Bruker (Camarillo, CA). Methyltriethoxysilane (MTES) was purchased from Acros Organics (NJ). 3-(Aminopropyl) triethoxysilane (APTES) and triisopropylsilane (TIPS) were bought from Sigma-Aldrich (Jerusalem, Israel). Ethanol absolute was acquired from the Gadot Group (Netanya, Israel). Triethylamine was purchased from Alfa-Aesar (Lancashire, UK). *N*-Diisopropylethylamine (DIEA), trifluoro acetic acid (TFA), *N*-methyl-2-pyrrolidone (NMP), dimethylformamide (DMF), chloroform, and piperidine were obtained from Bio-Lab Ltd. (Jerusalem, Israel). Fluorenylmethyloxycarbonyl-PEG-*N*-hydroxysuccinimide (Fmoc-PEG-NHS), 5000 Da, was purchased from Iris Biotech GmbH (Marktredwitz, Germany). Acetic anhydride was bought from Merck (Darmstadt, Germany). The protected peptides were purchased from GL Biochem (Shanghai, China). The protected peptides had an Fmoc protecting group at the N-terminus and protecting groups on the side chains (where required). Pentamethyl-2,3-dihydrobenzofuran-5-sulfonyl (Pbf) protected the side chain of arginine, *tert*-butyloxycarbonyl (Boc) protected the side chain of lysine, and tertiary butyl (*^t^*Bu) protected the side chain of serine. The free peptides were acquired from GL Biochem (Shanghai, China) or synthesized using solid-phase peptide synthesis (see the Supporting Information for details regarding peptide synthesis). Protected amino acids Fmoc-Lys(Boc)-OH, Fmoc-Ser(*^t^*Bu)-OH, Fmoc-Val-OH, Fmoc-Gly-OH, Fmoc-Met-OH, Fmoc-Pro-OH, and Fmoc-Arg(Pbf)-OH were obtained from GL Biochem (Shanghai, China). Coupling reagents HBTU and HCTU were bought from Luxembourg Bio Technologies Ltd. (Ness Ziona, Israel). The water used in this study was ultrapure deionized water (Milli-Q, Merck, Kenilworth, NJ) unless stated otherwise.

### Real-Time Adhesion Measurement Using Quartz Crystal Microbalance with Dissipation Monitoring (QCM-D)

Peptide adhesion to the hydroxyapatite (HAp) surface was monitored using QCM-D (QSense Explorer, Biolin Scientific, Gothenburg, Sweden). Experiments were performed as previously described^51^ with modifications. Briefly, HAp-coated QCM-D sensors (QSensor QSX 327 HA, Biolin Scientific) with a fundamental frequency of 5 MHz were used. Surface characterization of the sensors using AFM and X-ray photoelectron spectroscopy (XPS) is available in the Supporting Information (Figure S2, Table S1, Table S2, and SI results). Prior to each experiment, the sensors were cleaned according to the manufacturer’s procedure. All measurements were done in flow conditions using a digital peristaltic pump (IsmaTec Peristaltic Pump, IDEX) at a flow rate of 0.1 mL/min. The peptides were dissolved in Tris buffer (pH 7.2, 10 mM, 154 mM ionic strength adjusted using sodium chloride) up to a final concentration of 1.15 mM. The peptide solution was cycled into the flow cell for 18 h. The sensors were then washed with buffer.

### Adhesion under Different Medium Ionic Strengths

To determine whether the adhesive properties of the peptide are affected by the ionic strength of the buffer, the adhesion process of the native peptide was monitored using QCM-D with different solution ionic strengths. The measurements were performed as described above, with Tris buffer (pH 7.2, 10 mM) with different ionic strengths (154, 500, 750, 1000, and 2000 mM) adjusted using sodium chloride.

### QCM-D Data Analysis

All QCM-D curves were exported using QTools (Biolin Scientific), and the change in frequency was analyzed using Matlab (Mathworks, Natick, MA). The base frequency and time of each curve were aligned to the point of peptide addition. The change in frequency was calculated between the initial frequency and the frequency at the end of washing. For the ionic strength assays, the washing period was excluded from the frequency change calculation due to a high frequency change upon bulk changes. This means the frequency change was calculated for ionic strength assays between the point of injection and the end of adhesion after 18 h. For clarity of display, the data in the curves plotted were averaged and smoothed over a period of 4 min.

DFind (Biolin Scientific) was used to fit each curve with the Voigt viscoelastic model.^30,31^ A peptide film density of 1100 kg L^–1^ was used to model all measurements as previously described;^52,53^ this density corresponds to hydrated protein thin films. The surface density was exported and further analyzed using Matlab. For each measurement, the change in surface density was calculated for each period separately (i.e., layer buildup and washing periods) and summed to calculate the final layer density obtained in each measurement. The surface concentration was then calculated by dividing the surface density by the molecular weight of the corresponding peptide sequence.

### Adhesion Measurement Using Single-Molecule Force Spectroscopy (SMFS)

To assess the adhesion forces between the peptide and HAp, single-molecule force spectroscopy (SMFS) was performed using a Nanowizard 3 (JPK BioAFM, Berlin, Germany) atomic force microscope (AFM).

### SMFS Probe Functionalization

The probes were functionalized with the peptide as previously described.^24,42^ Briefly, silicon nitride cantilevers with silicon tips with a nominal tip radius of 2 nm and nominal spring constant ranging from 0.01–0.6 N/m were cleaned by dipping in ethanol for 20 min. They were then dried at room temperature and treated with O_2_ plasma (Atto, Diener Electronic, Ebhausen, Germany) for 5 min. Next, the cantilevers were suspended above (3 cm) a solution containing APTES and MTES in a ratio of 1:15 (v/v). The silane mixture and suspended probes were placed under nitrogen atmosphere within a desiccator and connected to a vacuum pump. The reaction took place over 2 h under vacuum to allow the formation of a monolayer. The probes were then heated to 75 °C for 10 min to dry and cooled back to room temperature. The tips were then immersed in a solution of Fmoc-PEG-NHS (Mw 5000 Da) at a concentration of 5 mM and 0.5%_v/v_ triethylamine in chloroform for 1 h at room temperature. This was followed by extensive washing with chloroform and DMF. The Fmoc protecting group was removed from the PEG linkers by dipping in 20%_v/v_ piperidine in DMF for 30 min, followed by extensive washing with DMF and NMP. Next, the desired peptide was coupled to the PEG linker via an amide bond between the carboxylic terminus of the peptide and the amine end of the PEG. This was achieved by dipping the probes in 5 mL of coupling solution containing 10 mg of fully protected peptide, and an equivalent amount of DIEA and HBTU in NMP for 2 h. The probes were then washed extensively by dipping in NMP. All amine groups that did not react were protected by the acetyl group. Acetylation was done by dipping the tips in a solution containing 67.5 μL of DIEA and 147 μL of acetic anhydride in 1.5 mL of NMP. The tips were then washed with NMP and DMF. Next, the Fmoc group was removed from the peptide N-terminus by dipping 20%_v/v_ piperidine in DMF for 30 min, followed by extensive washing with DMF and chloroform. Finally, the side groups of the peptide were deprotected by treating the tips with a solution containing 95% TFA, 2.5% TIPS, and 2.5% water for 1 h. The functionalized probes were extensively washed with chloroform, DMF, ethanol, and ultrapure water and dried overnight in a vacuumed desiccator.

### SMFS Substrate Preparation

For SMFS measurements, HAp tablets were purchased from Clarkson Chromatography (Williamsport, PA). To reduce the possibility of forming multiple bonds between the functionalized tip and the substrate, due to the high surface roughness, these substrates were polished using a Saphir 520 Grinder-Polisher (QATM, Salzburg, Austria). The substrates were washed with water and stored in a vacuumed desiccator until use.

### SMFS Measurements

The spring constant of each cantilever was calibrated prior to measurement using the thermal fluctuation method (included in the AFM software) with an absolute uncertainty of approximately 10%.^54^ Measurements were performed in Tris buffer. The functionalized probes were approached to a polished HAp substrate up to contact with an applied force of approximately 200 pN. The cantilever was then retracted at various loading rates until complete separation from the substrate with an overall distance of 500 nm.

### SMFS Data Analysis

All acquired force versus distance curves were automatically analyzed using ForSDAT^44^ (the sourcecode of version 1.1 is available for download from https://github.com/TaDuAs/ForSDAT). See the Supporting Information for the ForSDAT analysis configuration files.

Detachment forces, as well as apparent loading rates, were calculated using the wormlike chain (WLC) model.^55−58^ Specific interactions were detected using the smoothing peak rupture association method.^44^ Experiments wielding specific interactions in less than 4% for the native peptide and less than 2.5% for K7A of the curves were discarded under the assumption that there was a problem with the tip functionalization process, or it was degraded.

### Peptide–HAp Interactions Using Molecular Dynamics (MD) Simulation

To investigate the binding sites and conformation of the peptide during the interaction with the HAp surface, MD simulations were performed.

The molecular structures of the three peptides, the native peptide, K7A, and R11A, were created through *GaussView 6*,^59^ while the structure of the HAp slab was downloaded from the Cambridge Structural Database (CSD).^60^ The topologies of the peptides molecules and the HAp slab were created by the *GROMACS* 2019.6 package.^61^ The HAp slab was modeled according to a previous report.^22^ The CHARMM27 force field was applied for the simulations of the systems with the atomic charge, ε, and σ of each atom in the HAp slab from Hauptmann et al.,^62^ and the particle mesh Ewald (PME) method^63^ was applied for calculations of long-distance electrostatic interactions. The LINCS algorithm was employed to constrain all of the covalent bond lengths.^64^ According to the previous works^22^ and our preliminary simulations, the peptides tended to interact with the (100) face of the HAp slab (Figure S10), to therefore give a periodic box of the size 6.933 × 3.869 × 20.575 nm^3^, where the (100) face of the HAp slab lay in the *XY*-plane. For the CHARMM27 force field, TIP3P water molecules^22^ were filled into the boxes, giving the systems of 1 peptide molecule, 1 HAp slab, and 15 892, 15 887, 15 896, and 15 929 water molecules for the three systems, respectively. Because the native peptide, K7A, and R11A carried net charges of +2e, +e, and +e, respectively, 2, 1, and 1 Cl^–^ ions were added to neutralize the corresponding systems. After neutralization, energy minimization was performed with the steepest descent algorithm for each of the three systems. The system then was equilibrated at *T* = 298.15 K for 0.1 ns, where a Berendsen thermostat was used, respectively. Subsequently, MD simulations were performed for the three systems at *T* = 298.15 K for 50 ns with a Berendsen thermostat, respectively. After the simulation of 50 ns, each peptide was found to be adsorbed at the (100) face of the HAp slab, and the peptide was pulled away from the HAp surface with a constant velocity (0.01 nm per ps) through SMD simulation. In both of the MD and SMD simulations, short-ranged electrostatic and van der Waals interactions between any two atoms were cut off if the atomic distance reached 1.4 nm. During the SMD simulations, the distance between the center of mass (COM) of the peptide and that of the HAp slab as well as the corresponding pulling force were recorded, which provided straightforward information on the adsorptions of the peptides on the HAp surface. All of the snapshots and videos of the systems were rendered through VMD software.^65^

## Supporting Information Available

Supporting figures for QCM-D analysis, QCM-D characteristic curves, QCM-D sensor characterization, FT-IR results, analysis and method, secondary structure analysis results and method, supporting material for MD simulations, and SPPS method (PDF)
Supporting video S1 of the SMD simulation of the native peptide (AVI)
Supporting video S2 of the SMD simulation of K7A (AVI)
Supporting video S3 of the SMD simulation of R11A (AVI)
ForSDAT analysis configuration files (ZIP)

## Supplementary Material

la1c02293_si_001.pdf

la1c02293_si_002.avi

la1c02293_si_003.avi

la1c02293_si_004.avi
