# Supplementary material for: Understanding the Adhesion Mechanism of Hydroxyapatite-Binding Peptide
Source: Langmuir. 2022 Jan 7;38(3):968–78. doi: 10.1021/acs.langmuir.1c02293 (PMC8793143; doi:10.1021/acs.langmuir.1c02293)
Supplement: Supplementary file 1 — la1c02293_si_001.pdf [file la1c02293_si_001.pdf]

Supplementary Information

Understanding the Adhesion Mechanism of Hydroxyapatite-Binding Peptide

Tal Duanis-Assaf[1], Tan Hu[1,2], Maayan Lavie[1], Zhuo Zhang[2], Meital Reches[1]*

[1] Institute of Chemistry and The Center for Nanoscience and Nanotechnology, The Hebrew University of Jerusalem, Jerusalem 91904, Israel

[2] College of Food Science and Technology, Huazhong Agricultural University, Wuhan, Hubei 430070, PR China; Key Laboratory of Environment Correlative Dietology, Huazhong Agricultural University, Ministry of Education, Wuhan, Hubei 430070, PR China

EXPERIMENTAL INFORMATION

Peptide synthesis

Peptides were synthesized using Fmoc solid-phase peptide synthesis (SPPS). The peptides were synthesized on preloaded TentaGel S Trityl resin (Rapp Polymere GmbH, Germany) as described elsewhere[1].

Peptide purity was determined using Alliance analytical HPLC (Waters, Milford, MA) and an electrospray ionization LCQ Fleet Ion Trap mass spectrometer (Thermo Scientific, Waltham, MA) as previously described[1].

Determination of peptide structure in solution using Fourier-transform infrared (FT-IR) spectroscopy

To determine the peptide structure, FT-IR spectroscopy was performed using a Nicolet 6700 FT-IR spectrometer mounted with a deuterated triglycine sulfate (DTGS) detector (Thermo Fisher Scientific, MA, USA). Peptides were dissolved in Tris buffer (pH 7.2, 10mM, 154mM ionic strength adjusted using sodium chloride) prepared in D_2O, to a final concentration of 1.15 mM. A drop of 10 μL peptide solution was sandwiched between two CaF_2 windows (Sigma-Aldrich, St. Louis, MO, United States) mounted with a PSA-M25 12 μm spacer (Harrick Scientific Products, Pleasantville, NY, United States). Measurements were performed with 4 cm^{-1} resolution and averaged over 2000 scans.

Spectra were analyzed using PeakFit software version 4.12 (Seasolve, San-Jose, CA, United States). Each spectrum was baseline subtracted and smoothed, then peaks were resolved using the second derivative method followed by iterative optimization to minimize residuals.

Determination of adhered peptide structure using Attenuated total reflectance (ATR) Fourier-transform infrared (FT-IR) spectroscopy

To determine the structure of the bound peptide, ATR FT-IR spectroscopy was performed using the same FT-IR instrument mounted with a VariGATR Ge-ATR setup (Harrick Scientific). The

experiments were performed on hydroxyapatite (HAp) coated QCM-D sensors, after peptide adhesion, as described in the main text. The measurements were conducted with an applied force of 350 N, an incident angle of 65°, 4 cm^{-1} resolution and averaged over 2000 repeats.

Spectra were analyzed using PeakFit software version 4.12 (Seasolve, San-Jose, CA, United States). Each spectrum was baseline subtracted and smoothed, then peaks were resolved using the second derivative method followed by iterative optimization to minimize residuals.

Topographic and roughness analysis of the QCM-D sensors using AFM.

To analyze the roughness and surface topography of the QCM-D HAp sensors before and after peptide adhesion, AFM imaging was performed using a Dimension XR (Bruker, Camarillo, CA) in tapping mode. Imaging of the clean sensors was done using RTESP-300 (Bruker) silicon tip (cantilever length 125 μm, nominal frequency 300 kHz). Imaging of peptide-coated sensors was done using RFESP-75 (Bruker) silicon tip (cantilever length 225 μm, nominal frequency 75 kHz). The surface roughness was calculated from the images over an area of 1 μm^2.

X-ray photoelectron spectroscopy (XPS) analysis

To evaluate the HAp layer on the QCM sensor surface, X-ray Photoelectron Spectroscopy (XPS) analysis was conducted using a Kratos AXIS Supra spectrometer (Kratos Analytical Ltd., Manchester, United Kingdom). Spectra were acquired using the Al-Kα monochromatic X-ray source (1,486.6 eV). The sample take-off angle was 90◦ (normal to the analyzer). The vacuum pressure in the analyzing chamber was maintained to $2 \cdot 10^{-9}$ Torr. High-resolution XPS spectra were collected for P 2p, Si 2p, Ca 2p, Ti 2p, O 1s peaks with pass energy 20 eV and 0.1 eV step size. Data analysis was done using ESCApe processing program (Kratos Analytical Ltd.) and Casa XPS (Casa Software Ltd.).

Secondary structure analysis

The secondary structures of the peptides were analyzed using DSSP[2] in terms of the locations of hydrogen bonds. Since the peptides were relatively small in the study, it was supposed that the peptides tended to form a helix, turn, or coil structure. In DSSP, an N-turn (normally N = 3, 4, or 5) represented a turn where the C=O group of the *i*-th residue has formed a hydrogen bond with the N-H group of the $(i+N)$-th residue in the peptide chain[3].

RESULTS

Analysis of FT-IR Spectroscopy

To gain insights into the peptide conformation, FT-IR analysis was conducted. Figure S4 shows the FT-IR spectrum of the native peptide as well as K7A and R11A. The spectra had a dominant peak at 1673 cm^{-1} for the native peptide and 1672 cm^{-1} for both K7A and R11A, which is a typical band for turns[4-5]. The native peptide spectrum showed minor peaks at 1620, 1637 and 1654 cm^{-1}. K7A and R11A both had two minor peaks with higher magnitude compared to the native peptide, at 1625 and 1646 cm^{-1} for K7A and 1619 and 1644 cm^{-1} for R11A. The 1619-1625 cm^{-1} band is possibly related to the formation of intermolecular β-sheets[6], however, it is also abundant in β-hairpin structures[7-8]. The 1644-1646 cm^{-1} band detected in K7A and R11A spectra are characteristic of disordered peptides. The 1637 and 1654 cm^{-1} bands of the native peptide could be related to β-sheets and either α-helix or turns respectively[4]. However, it is also possible that these bands are the result of disordered segments, similar to the mid-range peaks detected in the spectra of the other derivatives. It is worth mentioning that the β-turn band at 1672-1673 cm^{-1} was

the dominant feature in all spectra, and the other bands likely represent less stable conformations. Moreover, the peaks of non-structured segments and intermolecular β-sheet stacking were more abundant for K7A and R11A than in the native peptide, possibly indicating that the native peptide has a more stable β-turn structure than either K7A and R11A.

Further investigation of the structure of the bound peptide was done using ATR FT-IR. The spectra are shown in Figure S5. The ATR FT-IR spectrum of the native peptide had a wide peak. Analysis using the second derivative method revealed two peaks at 1625 cm^{-1} and 1667 cm^{-1}. These peaks may be related to the 1620 cm^{-1} and 1673 cm^{-1} peaks observed in solution FT-IR. However, they are shifted, possibly due to interaction with the surface[9] or with other bound peptide molecules[10]. Moreover, the 1625 cm^{-1} peak is roughly the same magnitude as the 1667 cm^{-1} peak, possibly indicating a change in the abundance of both structures.

X-ray photoelectron spectroscopy (XPS) analysis

XPS analysis reveals the presence of calcium and phosphate in a Ca:P ratio of 1.5. This is within the error margin from the expected 5:3 ratio of HAp. The sensor bulk material is quartz. Titanium is used as an adhesive layer for the HAp nanoparticles. Both titanium and silicone were detected at abundant quantities. The oxygen abundance is probably due to quartz and oxides in the coating.

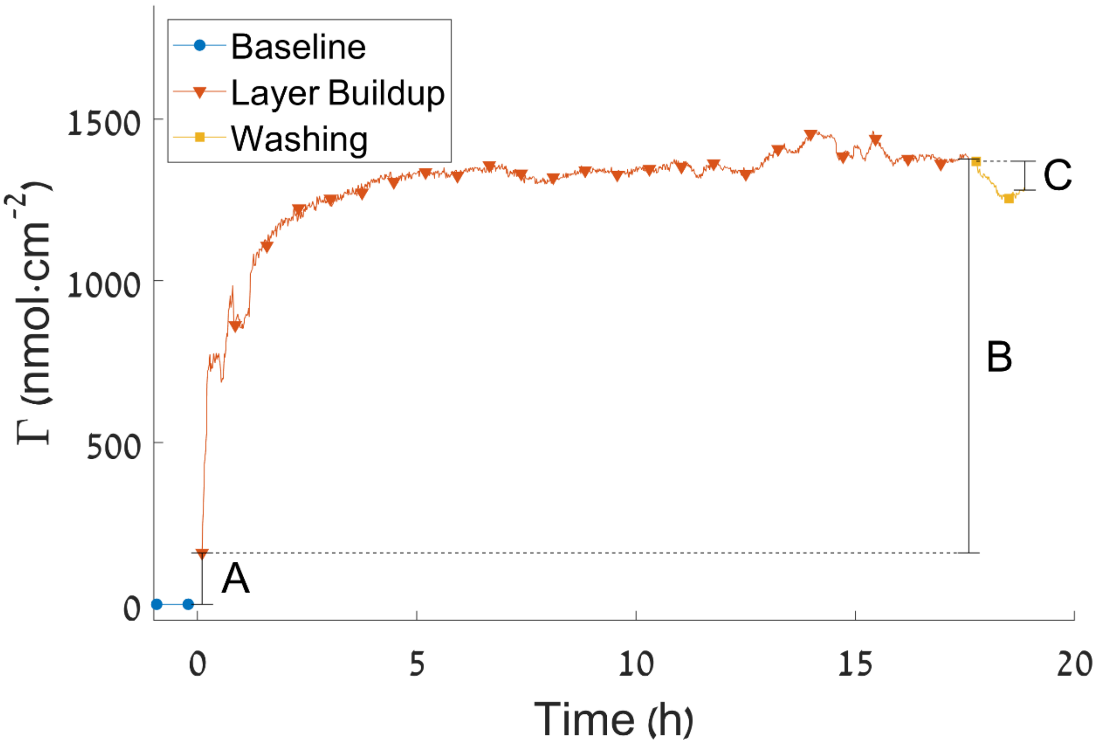

Figure S1. Example of area density propagation over time obtained by fitting the Voigt model to a QCM-D adhesion curve. Each of the three plots represents a distinct period in the adhesion experiment cycle, starting with baseline, followed by layer buildup and final washing period. The overall change in area density is calculated by summing the change in area density during each period, visualized as lines B and C for layer buildup and washing periods respectively. Changes in the mechanical properties of the solution may cause abrupt changes in frequency[11]. These frequency jumps are also carried over to the modeling stage and are reflected by considerable changes in the calculated area density of the layer between periods, represented here by line A. Such jumps in area density between periods were neglected from the calculation of the overall adhered peptide density, under the assumption that they mostly reflect changes in the solution mechanical properties rather than actual changes in layer thickness.

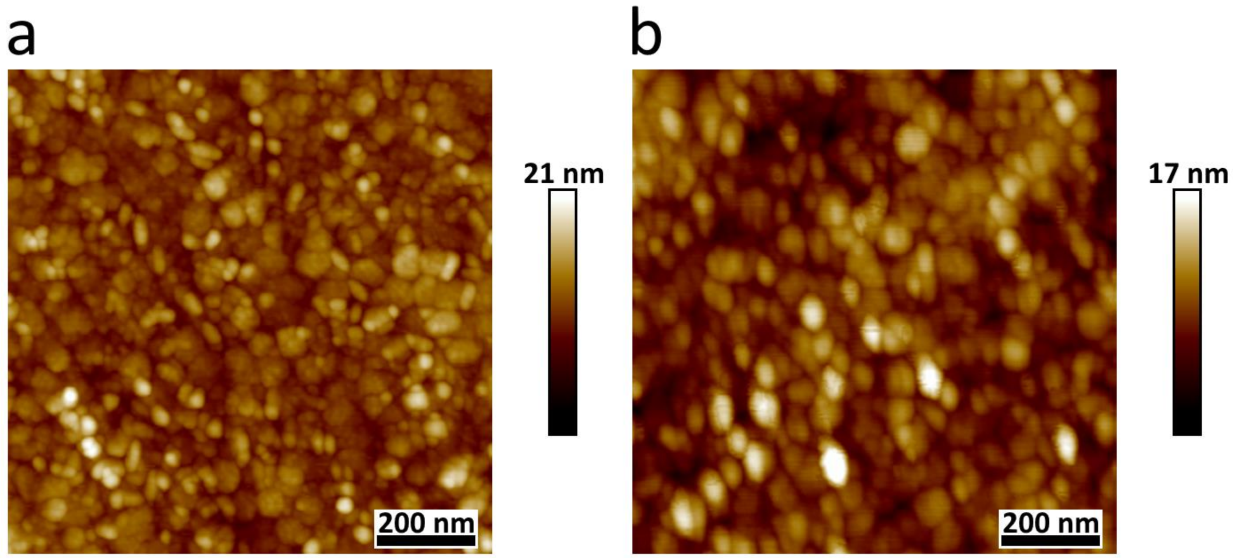

Figure S2. Typical AFM topographic images of the HAp QCM-D sensor. (a) Clean sensor surface. (b) Sensor surface after adhesion measurement with the native peptide.

Table S1. Surface Roughness of QCM-D sensors measured using AFM, prior to and after adhesion measurement with the native peptide.

	**Rq (nm)**	**Ra (nm)**	**Rmax (nm)**
**Clean HAp sensor**	1.83	1.43	15.4
**Native peptide**	2.04	1.57	17.4

Rq – RSMD roughness. Ra – Average roughness. Rmax – Maximal peak-to-valley difference.

Table S2. X-ray photoelectron spectroscopy (XPS) analysis of the QCM-D sensor surface.

	**Atomic conc. [%]**	**Error [%]**
**P 2p**	0.64	0.07
**Si 2p**	6.9	0.3
**Ca 2p**	0.96	0.04
**Ti 2p**	23.3	0.2
**O 1s**	68.2	0.3

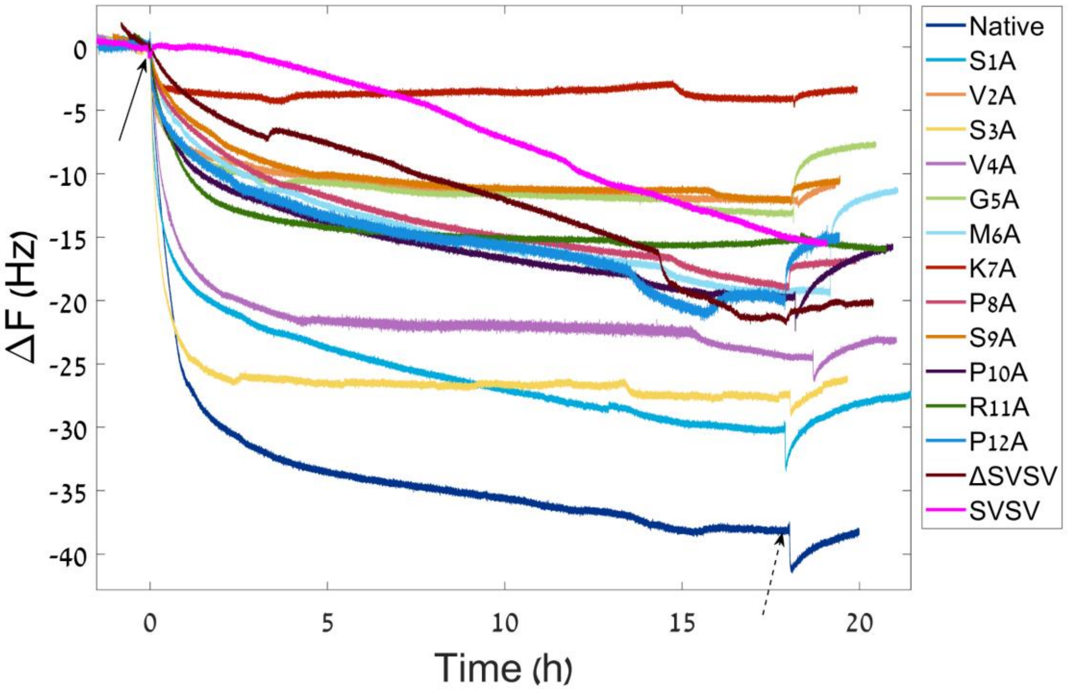

Figure S3. QCM-D real-time adhesion curves of the native peptide and all derivatives. Time 0 is aligned to the time of peptide injection and the adhesion is measured over 18 h followed by a period of washing. The arrows show the point of peptide injection (continuous line) and the start of the washing period (dashed line).

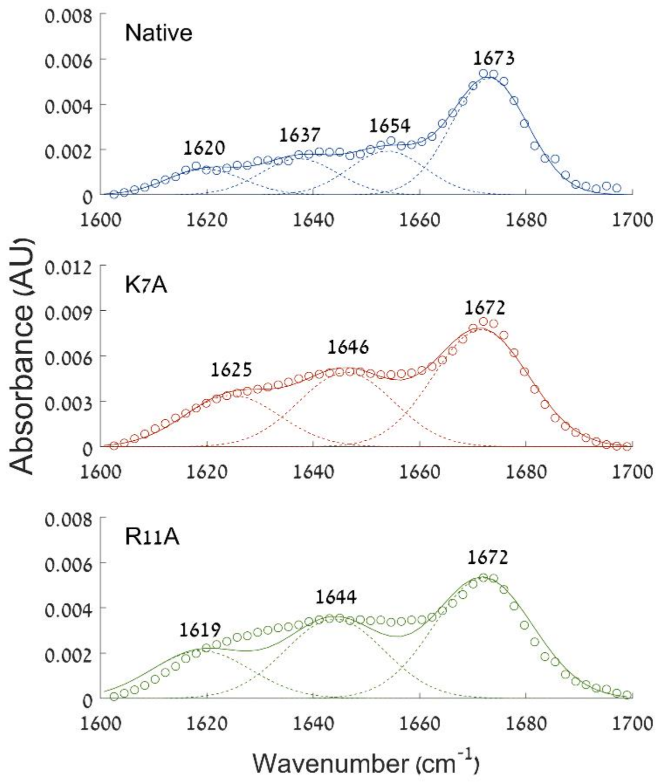

Figure S4. FT-IR spectroscopy of the native peptide, K7A and R11A. The circles represent the acquired spectra, the dotted lines represent peaks identified by the second derivative analysis, and the continuous lines represent the sum of detected peaks.

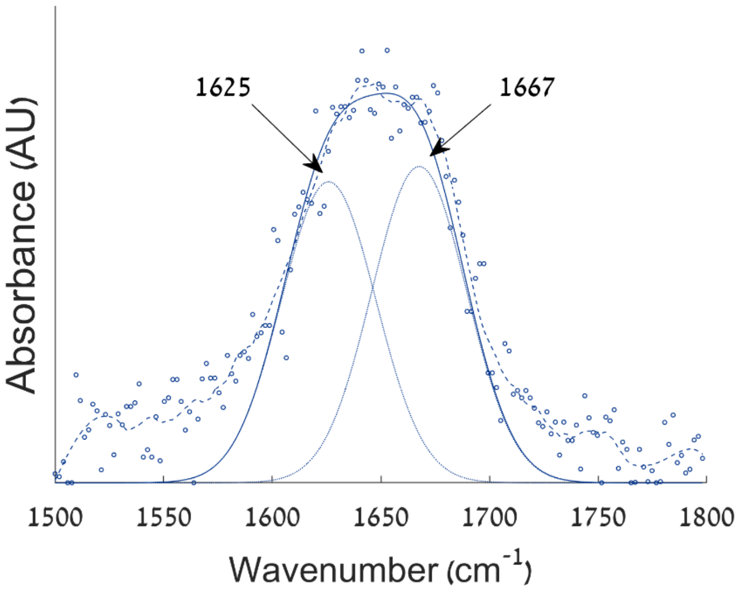

Figure S5. ATR FT-IR spectroscopy of the native peptide. The circles represent the acquired spectrum, the dashed line represents the smoothed signal, the dotted lines represent peaks identified by the second derivative analysis, and the continuous lines represent the sum of detected peaks.

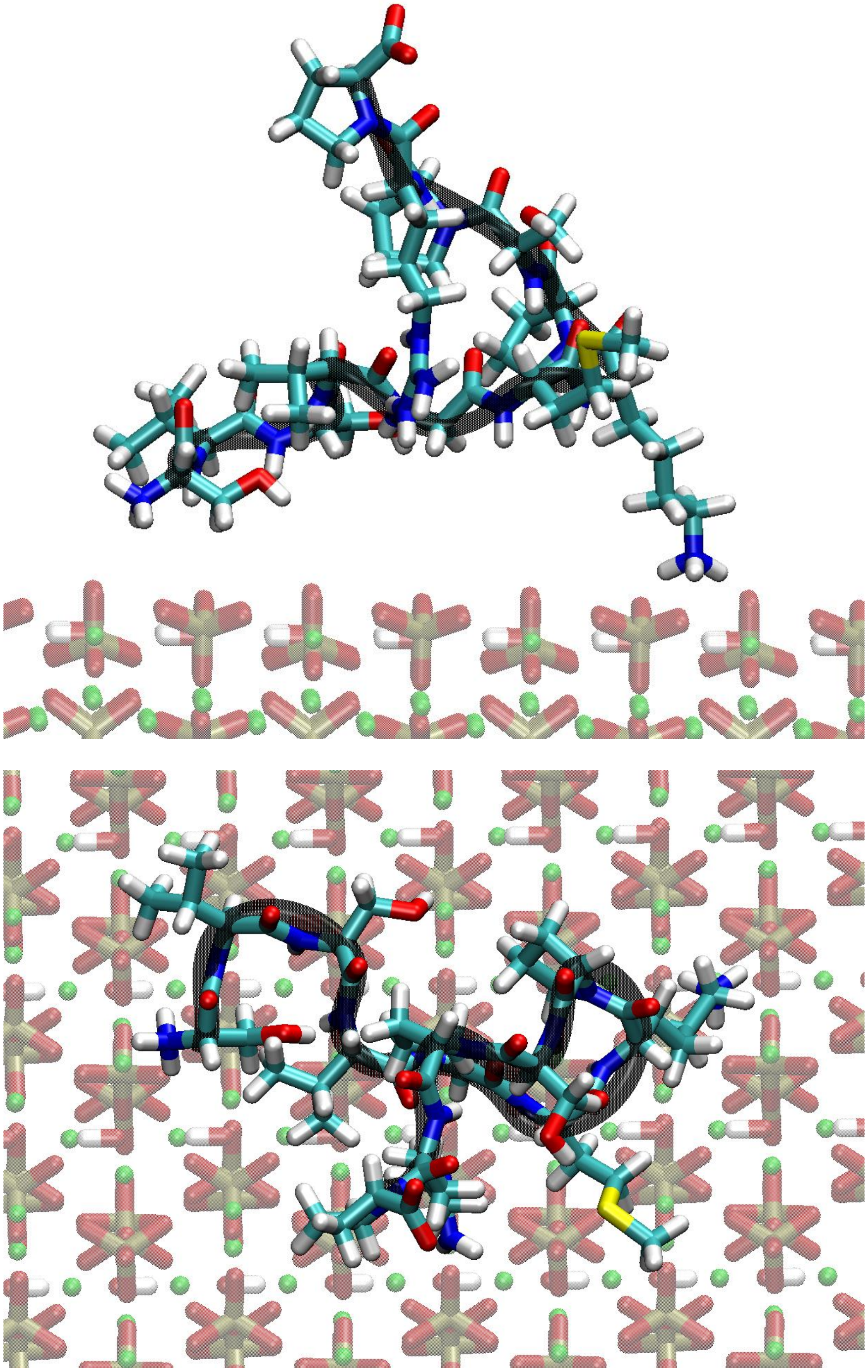

Figure S6. Snapshots of the native peptide conformation over the HAp surface at the end of the molecular dynamics simulation. The black thread highlights the peptide backbone.

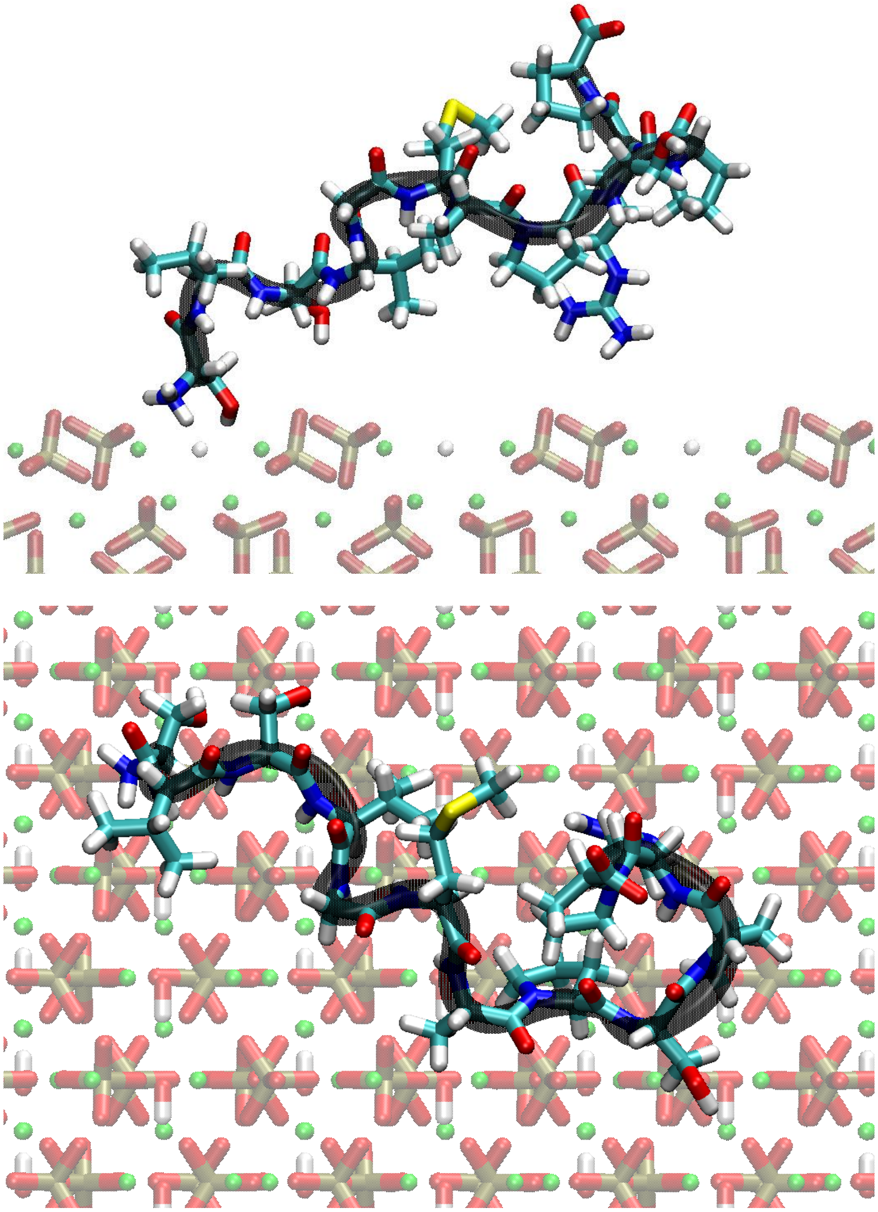

Figure S7. Snapshots of K7A peptide conformation over the HAp surface at the end of the molecular dynamics simulation. The black thread highlights the peptide backbone.

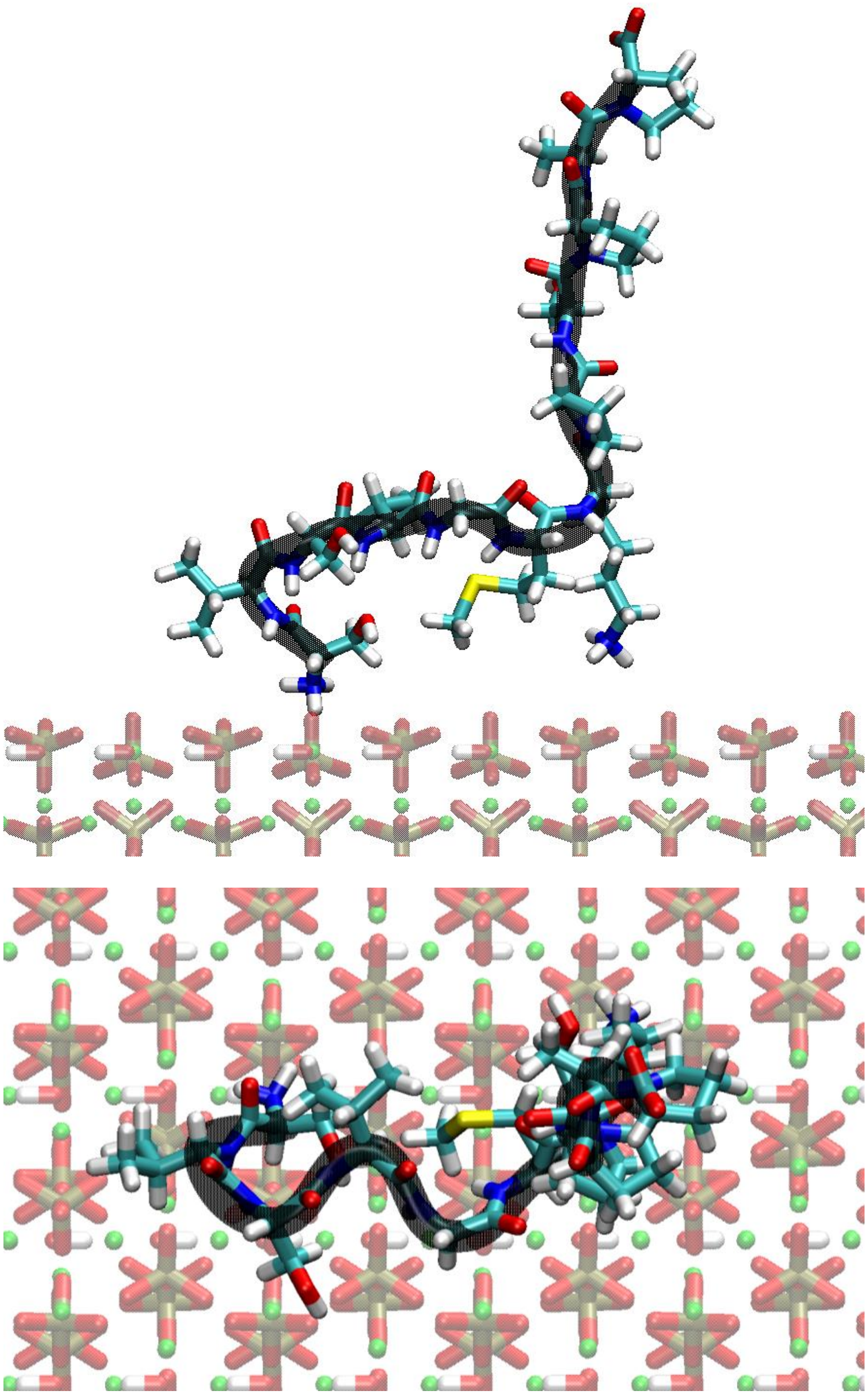

Figure S8. Snapshots of R11A peptide conformation over the HAp surface at the end of the molecular dynamics simulation. The black thread highlights the peptide backbone.

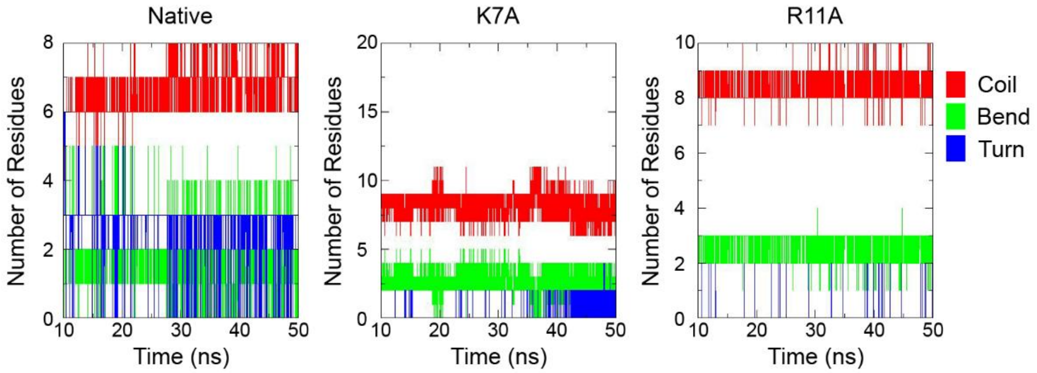

Figure S9. Secondary structure analysis of the native (a), K7A (b) and R11A (c) peptides. The plots show the number of residues that take part in different secondary structures in each time frame in the molecular dynamics simulation.

Table S3. Most prevalent structure per residue.

**#Residue**	**Native**		**K7A**		**R11A**	
**1**	S	Coil	S	Coil	S	Coil
**2**	V	Coil	V	Coil	V	Coil
**3**	S	Turn/Bend	S	Coil	S	Turn/Bend
**4**	V	Coil	V	Turn/Bend	V	Coil
**5**	G	Coil	G	Coil	G	Coil
**6**	M	Turn/Bend	M	Coil	M	Turn/Bend
**7**	K	Turn/Bend	A	Coil	K	Turn/Bend
**8**	P	Turn/Bend	P	Turn/Bend	P	Coil
**9**	S	Coil	S	Turn/Bend	S	Coil
**10**	P	Coil	P	Coil	P	Coil
**11**	R	Coil	R	Coil	A	Coil
**12**	P	Coil	P	Coil	P	Coil

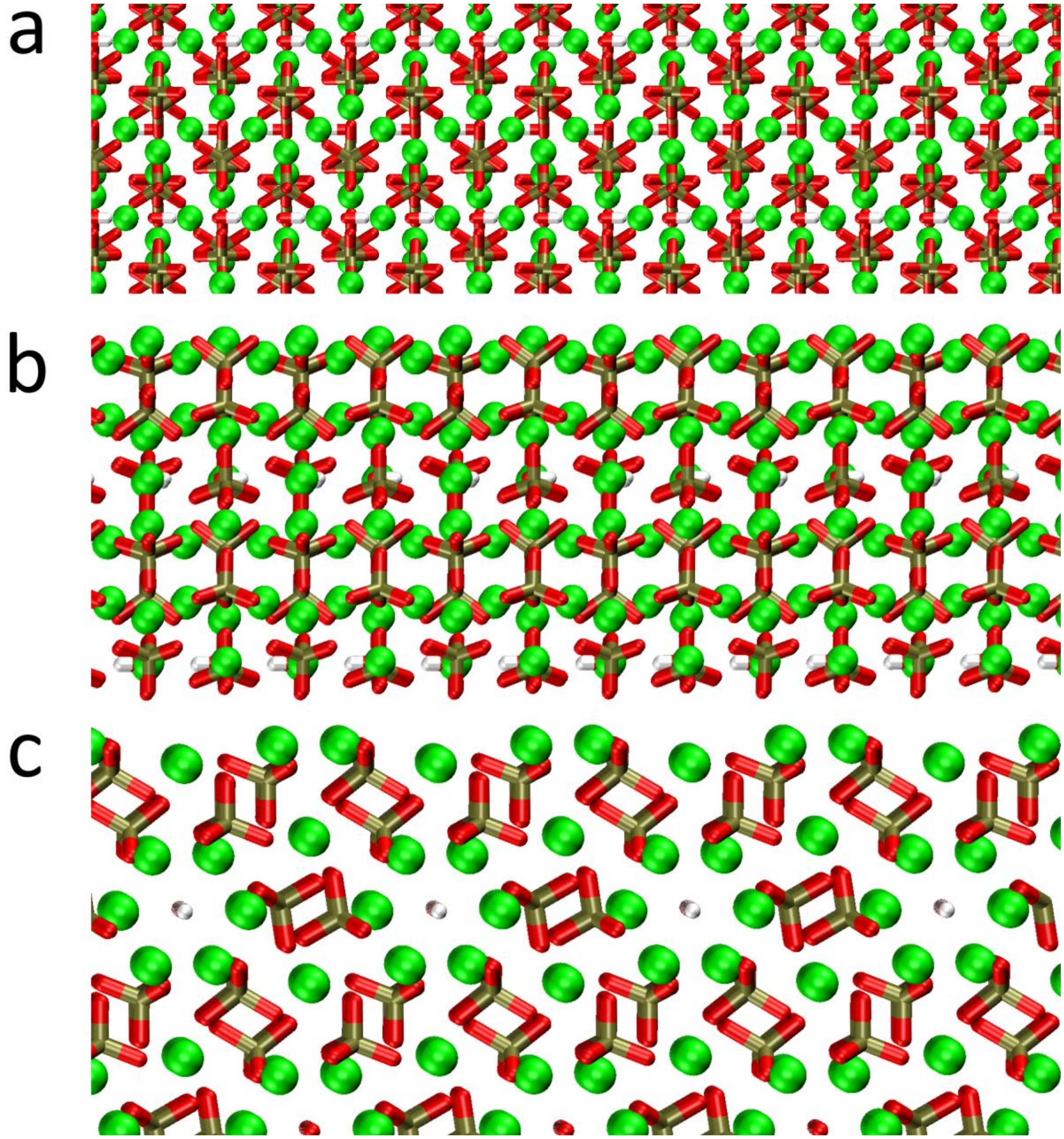

Figure S10. HAp slab crystal faces. (100) crystal face (a). (001) crystal face (b). (010) crystal face (c). The red, bronze, green and white represent oxygen, phosphoros, calcium and hydrogen atoms respectively.

SI REFERENCES

1. Yuran, S.; Dolid, A.; Reches, M., Resisting bacteria and attracting cells: Spontaneous formation of a bifunctional peptide-based coating by on-surface assembly approach. *ACS Biomater. Sci. & Eng.* **2018,** *4* (12), 4051-4061.
2. Kabsch, W.; Sander, C., Dictionary of protein secondary structure: pattern recognition of hydrogen-bonded and geometrical features. *Biopolymers* **1983,** *22* (12), 2577-2637.
3. Zhang, Z.; Hu, H.; Xu, X.; Pan, S.; Peng, B., Insights of Pressure-induced Unfolding of β-Lactoglobulin as Revealed by Steered Molecular Dynamics. *Adv. Theory Simul.* **2019,** *2* (6), 1800199.
4. Barth, A., Infrared spectroscopy of proteins. *Biochim. Biophys. Acta Bioenerg.* **2007,** *1767* (9), 1073-1101.
5. Goormaghtigh, E.; Cabiaux, V.; Ruysschaert, J.-M., Determination of soluble and membrane protein structure by Fourier transform infrared spectroscopy. *Physicochemical methods in the study of biomembranes* **1994**, 405-450.
6. Arrondo, J. L. R.; Goñi, F. M., Structure and dynamics of membrane proteins as studied by infrared spectroscopy. *Prog. Biophys. Mol. Biol.* **1999,** *72* (4), 367-405.
7. De Leon-Rodriguez, L. M.; Park, Y.-E.; Naot, D.; Musson, D. S.; Cornish, J.; Brimble, M. A., Design, characterization and evaluation of β-hairpin peptide hydrogels as a support for osteoblast cell growth and bovine lactoferrin delivery. *RSC Adv.* **2020,** *10* (31), 18222-18230.
8. De Leon-Rodriguez, L. M.; Hemar, Y.; Mitra, A. K.; Brimble, M. A., Understanding the metal mediated assembly and hydrogel formation of a β-hairpin peptide. *Biomater. Sci.* **2017,** *5* (10), 1993-1997.
9. Kowalczuk, D.; Pitucha, M., Application of FTIR method for the assessment of immobilization of active substances in the matrix of biomedical materials. *Materials* **2019,** *12* (18), 2972.
10. Cote, Y.; Nominé, Y.; Ramirez, J.; Hellwig, P.; Stote, R. H., Peptide-protein binding investigated by far-ir spectroscopy and molecular dynamics simulations. *Biophys. J.* **2017,** *112* (12), 2575-2588.
11. Feldoto, Z.; Pettersson, T.; Dedinaite, A., Mucin-electrolyte interactions at the solid-liquid interface probed by QCM-D. *Langmuir* **2008,** *24* (7), 3348-3357.
